# Dietary Intake of Minerals and the Risk of Ischemic Stroke in Guangdong Province, China, 2007-2008

**Published:** 2011-02-15

**Authors:** Wenbin Liang, Andy H. Lee, Colin W. Binns

**Affiliations:** Curtin University of Technology, School of Public Health and National Drug Research Institute, Perth, Western Australia, Australia; School of Public Health and National Drug Research Institute, Curtin Health Innovation Research Institute, Curtin University of Technology; Curtin University of Technology, School of Public Health and National Drug Research Institute, Perth, Western Australia, Australia

## Abstract

**Introduction:**

Previous studies have investigated the association between mineral intake and the risk of ischemic stroke, but results are inconsistent. We conducted a case-control study to ascertain the associations between intake of dietary potassium, calcium, magnesium, sodium, and iron and the ischemic stroke risk in the southern Chinese population.

**Methods:**

Information on lifestyle and typical food consumption was obtained from 374 hospital inpatients with ischemic stroke and 464 hospital-based control patients. Added sodium from salt or soy sauce could not be reliably quantified, but participants were asked to characterize their diet as low in salt, normal, or high in salt. Logistic regression analyses were performed to assess possible associations between the effects of mineral intake and ischemic stroke risk.

**Results:**

The mean weekly intakes of potassium, calcium, magnesium, and iron were lower among case patients than among control patients. Sodium was an exception. Lower stroke risk was associated with higher weekly dietary calcium or magnesium levels (adjusted odds ratio, 0.32) for the highest versus lowest category of intake, and significant dose-response relationships were seen. No significant associations were found for potassium, iron, or sodium. However, patients who consumed a salty diet were more than twice as likely as those whose diet was light in salt to experience an ischemic stroke.

**Conclusion:**

The findings suggest that lowering sodium intake while maintaining high levels of dietary calcium and magnesium may help prevent ischemic stroke in southern Chinese adults.

## Introduction

Stroke is one of the leading causes of disability and death in many countries across all levels of economic development. An estimated 15 million new cases of stroke occur worldwide annually. Stroke contributes to approximately 10% of total deaths globally (1), and the incidence of stroke is likely to increase during the next 20 years (2). Ischemic stroke accounts for more than 70% of all stroke cases in Western countries and approximately 60% of cases in China (3,4).

Various foods and nutrients are associated with the risk of stroke (5). A diet rich in potassium, calcium, and magnesium may reduce the risk of stroke, whereas decreasing sodium intake can lower blood pressure and stroke risk (6-8). The effect of iron intake is less clear (9). Because minerals are essential elements for the human body, epidemiologic studies have investigated the relationship between ischemic stroke and dietary mineral consumption, but the results are inconsistent (9-17). Moreover, to our knowledge, no published studies have investigated this issue in the southern Chinese population. In view of the limited and emerging evidence, we aimed to ascertain whether the dietary intake of these minerals is associated with the risk of ischemic stroke for southern Chinese adults. The study was part of a research project assessing the role of nutrition and lifestyle factors for the prevention of this major chronic disease.

## Methods

### Study population

Surveys for this case-control study were performed in the Guangdong Province of southern China from July 2007 through July 2008. Details of the methods have been described elsewhere (18). We recruited patients from 3 teaching hospitals in Foshan: the First People's Hospital of Shunde, First People's Hospital of Nanhai, and Second People's Hospital of Foshan. Cases were incident ischemic stroke patients referred from the inpatient wards of the hospital neurology departments; control patients were recruited from outpatient clinics of the departments of gastroenterology, dermatology, Chinese medicine, urology, and otolaryngology. All participants must have resided in Foshan for at least the past 5 years to be eligible. Selection criteria for ischemic stroke cases were sudden onset of a focal neurological event with symptoms lasting for more than 24 hours, subsequent confirmation of infarction in the brain by computed tomography or magnetic resonance imaging scans, and no history of stroke. Therefore, we considered only patients with a first-ever ischemic stroke (thrombotic or embolic). Eligible control patients had neither history nor clinical evidence indicating a previous stroke, and their treatment at the outpatient department was not related to any cardiovascular disease, a malignant tumor, or diabetes. We also excluded patients who had a diagnosis of Alzheimer disease or who had been on long-term diet modification for medical reasons. The hospital-based control patients were matched by frequency to stroke patients within 5 years of age and were recruited during the same period as stroke patients. The primary author (W.L.) initially approached 500 incident stroke patients and 600 eligible control patients; 374 case patients and 464 control patients signed the consent form and completed the interview. No significant differences were found in mean age and sex distribution between participants and those who declined or subsequently withdrew from the study.

### Interview

The neurology departments of the 3 hospitals notified the primary author (W.L.) within 2 days after each admission for stroke. The primary author then arranged an interview soon after, before the patient was discharged from the hospital. The primary author approached control patients at random and recruited and interviewed them while they were waiting for medical service at outpatient clinics. All participants were assured of confidentiality and were advised of their right to withdraw from the project at any time without prejudice before giving formal consent. Each interview took an average of 45 minutes to complete. The human research ethics committee of Curtin University of Technology approved the protocol (approval no. HR 17/2007), and the participating hospitals granted access to medical records.

### Measurement of exposure

Information on typical food consumption and demographic and lifestyle characteristics was collected from the interview by using a structured questionnaire that had been specifically developed and validated for the southern Chinese population (19,20). The information collected included age, sex, weight, height, education level, smoking status and cumulative smoking (pack-years), alcohol consumption, and physical activity. When patients were unable to provide answers because of the effects of the stroke, the answers were obtained from their next of kin. The validity and reliability of using such proxy information have been established in previous studies (21-24). We used medical records to confirm self-reported height and weight measurements and medical history (presence of hypertension, hyperlipidemia, or diabetes) when these were available (approximately 70% of the time).

The semi-quantitative food frequency component of the questionnaire included 125 items covering foods commonly consumed in southern China and recorded both frequency and amount of consumption. The reference recall period was set at 1 year before the interview. We obtained the quantity of minerals for each food item from Chinese food composition tables (25). The minerals available for analysis from the nutrient database were iron, sodium, potassium, calcium, and magnesium. For each participant, we derived the dietary intake of each mineral per week by adding the corresponding food items and multiplying the standard serving size (in grams) by frequency of consumption. We similarly estimated weekly total energy intake (kcal) by summing the energy intake for individual food items. Sodium consumption from salt and soy sauce added to foods during cooking was difficult to quantify and thus was excluded in the calculation of sodium intake. To investigate the potential effect of salt, we asked the participants to rank their diet as low in salt, normal, or high in salt.

### Statistical analysis

We used descriptive statistics to characterize participants. After comparing the mineral intake patterns of case patients and control patients, we conducted unconditional logistic regression analyses to assess possible associations between specific dietary minerals and ischemic stroke risk, adjusting for total energy intake. We tested each quantitative mineral variable for linear trends and further categorized variables into quartiles on the basis of the empirical distribution among controls. The lowest intake level was the reference category in each model. Other independent variables included in the multivariable models were age, sex, body mass index (BMI), education level, cigarette smoking status, cumulative smoking (pack-years), alcohol drinking status, lifelong physical activity exposure, and presence of comorbidities. These variables were either established risk factors or plausible confounders (18). We used Stata version 10 (StataCorp LP, College Station, Texas) and set significance at *P* < .05.

## Results

The mean age (approximately 69 y) and BMI of case patients and control patients were similar (Table 1). The prevalence of hypertension, diabetes, and current smoking was higher among case patients than control patients. Case patients also had higher cumulative smoking exposure, and were less physically active over their life course than control patients.

Case patients' mean dietary intakes of potassium, calcium, and magnesium were significantly lower (*P* < .001) than those of control patients, whereas sodium intake in the 2 groups was similar. Although iron levels were higher among women in the control group than among women in the case group, the corresponding mean difference in men was not significant.

Stroke risk was inversely associated with higher weekly dietary calcium and magnesium levels (Table 2). The adjusted odds ratio (OR) for both minerals was 0.32 for the highest category of intake compared with the lowest category. No significant associations were found for potassium, iron, or sodium.

Sodium intake from the food assessment was positively associated with participants' self-rated sodium intake, which ranged from a mean (standard deviation [SD]) of 5,586 (SD, 3,998) mg/week to 7,967 (SD, 6,115) mg/week. Approximately one-third of stroke patients described their diet as high in salt. The stroke risk was more than twofold for Chinese adults on a diet high in salt compared with those who described their diet as low in salt, even after controlling for confounding demographic and lifestyle variables and total energy intake (Table 3). Proxy information was used in 22% of the cases.

## Discussion

We found inverse associations between ischemic stroke risk and dietary intake of calcium, magnesium, and potassium by southern Chinese adults, together with significant dose-response relationships for calcium and magnesium. An inverse association was also found for potassium but did not reach significance. However, regarding sodium intake, the risk of stroke more than doubled for study participants who self-reported a diet high in salt, suggesting the adverse effect of added salt and soy sauce during cooking.

High sodium intake has been linked to high blood pressure (26), unlike potassium, calcium, and magnesium, which have demonstrated beneficial effects (12,27-30). Nevertheless, previous results concerning the association between sodium intake and risk of stroke have been mixed (13,15,16). In addition to differences in sodium consumption levels, variations in underlying population characteristics, cooking methods, exposure measurements, and genetic disparity among studies also contribute to the inconsistencies.

The observed protective effects of calcium and magnesium appear to be consistent with findings from cohort studies conducted in the United States (10,14,31-33), Finland (15), and Japan (34). The role of potassium in stroke risk is unclear on the basis of our literature review (14,15,31,32).

Our results suggest that increased iron intake may elevate stroke risk. It has been hypothesized that higher iron stores could increase the risk of atherosclerotic cardiovascular disease (35). In contrast, a recent cross-sectional study involving 4 countries reported a significant inverse association between iron intake and blood pressure (36), whereas a randomized clinical trial observed no effect of reducing iron stores through phlebotomy on the risk of stroke and myocardial infarction after 6 years of intervention (37). Therefore, further investigation on the effect of dietary iron level is required.

This study has several strengths. It was the first to our knowledge to investigate the association between dietary mineral intake and ischemic stroke risk in southern China. The reference period for dietary exposure was set at 1 year before interview to avoid possible dietary change as a consequence of stroke. The sample size of 838 participants ensured sufficient power for the multivariable analysis. We included only first-ever ischemic stroke patients with diagnosis confirmed by brain image. All control patients were screened for stroke symptoms by a neurologist to ensure correct classification of their disease status. Moreover, the 3 participating hospitals served the entire Foshan catchment region so that our participants were representative of the target population.

Several limitations should be taken into account. First, salt consumption was difficult to quantify and was excluded in the calculation of sodium intake. Nevertheless, a salty diet was separately associated with a higher risk of stroke for Chinese adults, which supported the evidence based on dietary sodium intake. Second, only 5 major minerals derived from the Chinese diet were available from the nutrient database (25). Consequently, effects of other minerals such as phosphorus and zinc could not be evaluated.

Because of the retrospective study design, recall bias is possible. A major weakness concerned the accuracy of mineral intake. Our results were based on recall of dietary behaviors with a reference period of 12 months and relied on estimates of intake of dietary minerals that were not verified by using biomarkers. Although we assessed the typical diet using a validated and reliable food frequency questionnaire for the southern Chinese population, the self-reported information was subject to measurement errors. Face-to-face interviews were conducted in the presence of case patients' next-of-kin to improve interpretation and accuracy. Moreover, the primary author (W.L.) conducted all interviews to eliminate inter-interviewer bias. Control patients were recruited during the same period and from the same catchment area as were case patients. They should therefore provide comparable estimates of foods intake. But the inherent selection bias was unavoidable because of their voluntary participation in the study. Proxy information was used in 22% of the cases, yet we found no significant differences between index patients and their proxies on weekly intake of minerals. Information bias was unlikely, because the potential effects of dietary minerals have not been established in China and all participants were blinded to the study hypothesis. Nevertheless, residual confounding might be present even though we controlled for plausible confounding factors in the multivariable analyses.

The findings of this study suggest that lowering sodium intake while maintaining high levels of dietary calcium and magnesium may be beneficial to prevent ischemic stroke for Chinese adults. Prospective cohort studies that collect detailed dietary exposure information are recommended to confirm the observed effects of these minerals in other populations.

## Figures and Tables

**Table 1:** Characteristics of Participants by Sex and Ischemic Stroke Status,^a^ Guangdong Province, China, 2007-2008

Variable	**Men**		**Women**	

	**Case Patients, n = 226**	**Control Patients, n = 248**	**Case Patients, n = 148**	**Control Patients, n = 216**
**Mean age, y (SD)**	69.6 (8.0)	68.7 (7.0)	69.1 (9.2)	69.0 (9.0)
**Mean BMI, kg/m^2^ (SD)**	22.9 (2.7)	23.1 (3.0)	21.5 (3.6)	22.8 (3.6)
**Primary school only, n (%)**	104 (46.0)	88 (35.5)	82 (55.4)	103 (47.7)
**Hypertension, n (%)**	119 (52.7)	71 (28.6)	76 (51.4)	60 (27.8)
**Hyperlipidemia, n (%)**	51 (22.6)	23 (9.3)	17 (11.5)	36 (16.7)
**Diabetes, n (%)**	48 (21.2)	8 (3.2)	31 (20.9)	4 (1.9)
**Alcohol drinker, n (%)**	157 (69.5)	147 (59.3)	13 (8.8)	43 (19.9)
**Current smoker, n (%)**	162 (71.7)	139 (56.1)	15 (10.1)	11 (5.1)
**Cumulative smoking (pack-years)^b^ (SD)**	18.5 (22.1)	17.1 (22.2)	1.3 (6.8)	1.0 (5.5)
**Lifelong physical activity, n (%)**				
Never been involved	71 (31.4)	56 (22.6)	60 (40.5)	63 (29.2)
Previously but not anymore	84 (37.2)	53 (21.4)	39 (26.4)	49 (22.7)
Active just recently	12 (5.3)	15 (6.1)	10 (6.8)	19 (8.8)
Intermittently active	22 (9.7)	29 (11.7)	6 (4.1)	25 (11.6)
Always been involved	37 (16.4)	95 (38.3)	33 (22.3)	60 (27.8)
**Mean iron intake, mg/wk (SD)**	137 (51)	145 (49)	109 (38)	125 (50)
**Mean sodium intake,^c^ mg/wk (SD)**	7,319 (5,540)	7,270 (5,271)	5,973 (5,303)	6,055 (3,910)
**Mean potassium intake, mg/wk (SD)**	18,805 (8,107)	21,807 (8,908)	15,525 (5,852)	20,193 (9,820)
**Mean calcium intake, mg/wk (SD)**	3,379 (1,500)	4,165 (1,705)	2,969 (1,403)	3,765 (2,008)
**Mean magnesium intake, mg/wk (SD)**	1,921 (818)	2,260 (926)	1,576 (622)	2,068 (1,021)
**Total energy intake, kcal/wk (SD)**	17,286 (6,281)	16,923 (5,165)	12,403 (3,710)	13,957 (4,790)

Abbreviations: SD, standard deviation; BMI, body mass index.

a Ischemic stroke status defined as case patients (inpatients diagnosed with their first ischemic stroke and referred from the hospital neurology department) or control patients (outpatients recruited from the hospitals' clinics of gastroenterology, dermatology, Chinese medicine, urology, and otolaryngology).

b One pack-year of smoking is calculated by multiplying the number of packs of cigarettes smoked per day by the number of years the person has smoked.

c Excluding salt and soy sauce added during cooking.

**Table 2:** Weekly Dietary Mineral Intake and Ischemic Stroke Risk, Guangdong Province, China, 2007-2008

Mineral Intake, mg/wk^a^	Case Patients,^b^n (%)	Control Patients,^c^n (%)	Odds Ratio^d^(95% CI)	Adjusted Odds Ratio^e^ (95% CI)	*P* Value^f^
**Iron**					
≤100	114 (31)	116 (25)	1 [Reference]	1 [Reference]	.30
101-128	97 (26)	116 (25)	1.25 (0.79-1.97)	1.25 (0.75-2.09)	
129-160	95 (25)	116 (25)	1.91 (1.08-3.40)	1.94 (1.02-3.68)	
≥161	68 (18)	116 (25)	2.43 (1.06-5.58)	2.31 (0.91-5.86)	
**Sodium^g^**					
≤3,726	90 (24)	116 (25)	1 [Reference]	1 [Reference]	.49
3,727-5,565	92 (25)	116 (25)	1.39 (0.91-2.14)	1.34 (0.83-2.18)	
5,566-8,073	108 (29)	116 (25)	1.78 (1.13-2.82)	1.82 (1.09-3.06)	
≥8,074	84 (23)	116 (25)	1.35 (0.81-2.27)	1.30 (0.73-2.32)	
**Potassium**					
≤14,610	136 (36)	116 (25)	1 [Reference]	1 [Reference]	.06
14,611-19,614	123 (33)	116 (25)	0.94 (0.61-1.46)	0.92 (0.57-1.49)	
19,615-25,630	76 (20)	116 (25)	0.65 (0.36-1.17)	0.57 (0.30-1.09)	
≥25,631	39 (10)	116 (25)	0.45 (0.18-1.13)	0.46 (0.17-1.22)	
**Calcium**					
≤2,697	135 (36)	116 (25)	1 [Reference]	1 [Reference]	.003
2,698-3,759	142 (38)	116 (25)	1.06 (0.71-1.59)	0.97 (0.61-1.52)	
3,760-4,962	64 (17)	116 (25)	0.48 (0.28-0.80)	0.49 (0.28-0.88)	
≥4,963	33 (9)	116 (25)	0.28 (0.13-0.60)	0.32 (0.14-0.73)	
**Magnesium**					
≤1,478	131 (35)	116 (25)	1 [Reference]	1 [Reference]	.005
1,479-2,069	127 (34)	116 (25)	1.01 (0.65-1.57)	0.87 (0.54-1.42)	
2,070-2,668	83 (22)	116 (25)	0.74 (0.41-1.36)	0.58 (0.30-1.12)	
≥2,669	33 (9)	116 (25)	0.39 (0.15-1.01)	0.32 (0.11-0.91)	

Abbreviation: CI, confidence interval.

a For each mineral, we established 4 levels of intake, based on the quartiles of intake among the 464 control patients. We calculated mineral intake by using data from a food frequency questionnaire administered to both case patients and control patients.

b Case patients were inpatients diagnosed with their first ischemic stroke and referred from the hospital neurology department.

c Control patients were outpatients referred from the hospitals' outpatient clinics of gastroenterology, dermatology, Chinese medicine, urology, and otolaryngology.

d Odds ratios from separate logistic regression models adjusting for weekly intake of iron, sodium, potassium, calcium, and magnesium; weekly energy intake; sex; and age.

e Odds ratios from separate logistic regression models adjusting for weekly intake of iron, sodium, calcium, magnesium, and potassium; weekly energy intake; sex; age; body mass index; education level; lifelong physical activity involvement; smoking status; cumulative smoking (pack-years); alcohol drinking status; and presence of hypertension, hyperlipidemia, or diabetes.

f Based on likelihood ratio test.

g Excluding salt and soy sauce added during cooking.

**Table 3:** Risk of Ischemic Stroke,^a^ by Sodium Intake, Guangdong Province, China, 2007-2008

Sodium Intake^b^	Case Patients, n (%)	Control Patients, n (%)	Odds Ratio^c^ (95% CI)	Adjusted Odds Ratio^d^ (95% CI)
Low	26 (7)	95 (21)	1 [Reference]	1 [Reference]
Normal	224 (60)	227 (49)	3.88 (2.35-6.13)	2.47 (1.47-4.19)
High	121 (33)	139 (30)	3.34 (2.01-5.59)	2.33 (1.34-4.09)

Abbreviation: CI, confidence interval.

a Ischemic stroke status defined as case patients (inpatients diagnosed with their first ischemic stroke and referred from the hospital neurology department) or control patients (outpatients recruited from the hospitals' clinics of gastroenterology, dermatology, Chinese medicine, urology, and otolaryngology).

b Self-reported by study participants or their proxies.

c Odds ratios from logistic regression model adjusting for weekly energy intake, sex, and age.

d Odds ratios from logistic regression model adjusting for weekly energy intake, sex, age, body mass index, education level, lifelong physical activity involvement, smoking status, cumulative smoking (pack-years), alcohol drinking status, and presence of hypertension, hyperlipidemia, or diabetes.
